# In vitro CRISPR screening uncovers CRTC3 as a regulator of IFN-γ-induced ferroptosis of hepatocellular carcinoma

**DOI:** 10.1038/s41420-023-01630-8

**Published:** 2023-09-04

**Authors:** Li Li, Tao Xing, Yiran Chen, Weiran Xu, Bo Fan, Gaoda Ju, Jing Zhao, Li Lin, Cihui Yan, Jun Liang, Xiubao Ren

**Affiliations:** 1 Department of Immunology, Tianjin Medical University Cancer Institute and Hospital, National Clinical Research Center for Cancer, Tianjin, China; 2 Tianjin’s Clinical Research Center for Cancer, Tianjin, China; 3 Key Laboratory of Cancer Immunology and Biotherapy, Tianjin, China; 4 Department of Oncology, Peking University International Hospital, Beijing, China; 5 Key Laboratory of Carcinogenesis and Translational Research (Ministry of Education), Department of Oncology, Peking University Cancer Hospital and Institute, Beijing, China; 6 Department of Radiation Oncology, Fujian Medical University Cancer Hospital, Fujian Cancer Hospital, Fuzhou, China; 7 Department of Oncology, Beijing Tiantan Hospital, Capital Medical University, Beijing, China; 8 Beijing Chao-Yang Hospital, Beijing, China; 9 Department of Pathology and Neuropathology, University Hospital Tübingen, Tübingen, Germany

**Keywords:** Cancer therapeutic resistance, Cancer therapeutic resistance

## Abstract

Interferon-gamma (IFN-γ) exerts anti-tumor effects by inducing ferroptosis. Based on CRISPR/Cas9 knockout screening targeting genome-wide protein encoding genes in HepG2 and SK-Hep-1 cell lines, we found that cAMP response element-binding protein (CREB) regulated transcription coactivator 3 (CRTC3) protects tumor cells from drug-induced ferroptosis and significantly inhibits the efficacy of IFN-γ treatment in hepatocellular carcinoma (HCC). Mechanistically, CRTC3 knockout altered tumor cell lipid patterns and increased the abundance of polyunsaturated fatty acids (PUFAs), which enables lipid peroxidation and enhances the susceptibility of HCC cells to ferroptosis inducers. To scavenge for accumulated lipid peroxides (LPO) and maintain redox equilibrium, HCC cells up-regulate SLC7A11 and glutathione peroxidase 4 (GPx4) expressions to enhance the activities of glutamate-cystine antiporter (system xc−) and LPO clearance. As IFN-γ inhibiting system xc−, simultaneous treatment with IFN-γ disrupts the compensatory mechanism, and generates a synergistic effect with CRTC3 knockout to facilitate ferroptosis. Sensitizing effects of CRTC3 depletion were confirmed using typical ferroptosis inducers, including RSL3 and erastin. Sorafeinib, a commonly used target drug in HCC, was repeatedly reported as a ferroptosis inducer. We then conducted both in vitro and vivo experiments and demonstrated that CRTC3 depletion sensitized HCC cells to sorafenib treatment. In conclusion, CRTC3 is involved in the regulation of PUFAs metabolism and ferroptosis. Targeting CRTC3 signaling in combination with ferroptosis inducers present a viable approach for HCC treatment and overcoming drug resistance.

## Introduction

Globally, hepatocellular carcinoma is the sixth most prevalent cancer type, and the third leading cause of cancer-related deaths [1]. Due to late diagnosis, high recurrence rates and lack of efficient treatment options, prognostic outcomes for advanced HCC are extremely poor [1, 2]. Sorafenib was the first tyrosine kinase inhibitor to be approved for HCC treatment. According to the SHARP study, the objective response rate (ORR) of sorafenib was only 2.3% and it failed to prevent long-term progression [3]. Immune checkpoint inhibitors (ICIs) exhibit promising efficacies in HCC, however, drug resistance is still a challenge as the ORRs for mono drugs are less than 20% [4, 5]. ICIs activate CD8+ cytotoxic T lymphocytes (CTLs) to destroy tumor cells via granule exocytosis, apoptosis inducement, and cytokine secretion, especially IFN-γ [6]. Short-term exposure to IFN-γ positively modulates the tumor immune microenvironment (TIME) and facilitates the anti-tumor effect of CTLs [7]. In addition, IFN-γ can directly kill tumor cells by inducing ferroptosis in a cell signaling-dependent manner [8, 9]. Ferroptosis, a term that was first coined in 2012, is a unique form of programmed cell death that is characterized by iron-dependent accumulation of LPO [10, 11]. In cancer biology, ferroptosis modulates multiple processes, including the initiation, invasion, immunity and treatment resistance of cancers [12]. Compared to normal cells, tumor cells use higher levels of iron and lipid metabolites to facilitate their aggressive growth, which increases their susceptibility to ferroptosis [13]. Thus, ferroptosis induction is a promising approach for cancer treatment and overcoming drug resistance. IFN-γ was reported to promote ferroptosis by inhibiting system xc− and impairing LPO clearance [8]. Therefore, identifying tumor cells that are unfavorable for IFN-γ-induced ferroptosis might provide a clue for sensitizing HCC cells to treatment.

We conducted CRISPR/Cas9 knockout screening using HCC cells under stimulation of IFN-γ, and identified CRTC3 to be a critical mediator of IFN-γ resistance. CRTC3 is activated in response to 3’,5’-cyclic adenosine monophosphate (cAMP)-protein kinase A (PKA) signals, which regulate energy expenditure and lipid metabolism under hormonal and environmental stimuli [14, 15]. Lipid metabolism is closely associated with ferroptosis [16, 17]. LPO is produced from peroxidation of intracellular PUFAs-containing phospholipids, and the abundance of PUFAs determines the susceptibility of cells to ferroptosis. IFN-γ interacts with arachidonic acid (AA) to induce tumor cell ferroptosis [18]. We found that CRTC3 knockout promoted lipid peroxidation by increasing the abundance of PUFAs, including adrenic acid (AdA) and AA. To scavenge for redundant LPO and maintain redox homeostasis, HCC cells upregulated SLC7A11 (a key subunit of system xc−) and GPx4 expressions to enhance glutathione (GSH) synthesis and LPO clearance. As IFN-γ inhibited system xc− [8], it disrupted the compensatory mechanism and generated a synergistic effect with CRTC3 knockout to facilitate ferroptosis. Moreover, we confirmed the sensitizing effects of CRTC3 knockout to other ferroptosis inducers, including RSL3, which targets GPx4, as well as erastin and sorafenib, that target system xc−. Therefore, CRTC3 has a potential role in the regulation of PUFAs metabolism as well as ferroptosis and is a potential therapeutic target for HCC.

## Results

### CRISPR/Cas9-based screening identified CRTC3 to be a critical mediator of IFN-γ resistance in HCC cells

**Fig. 1:**
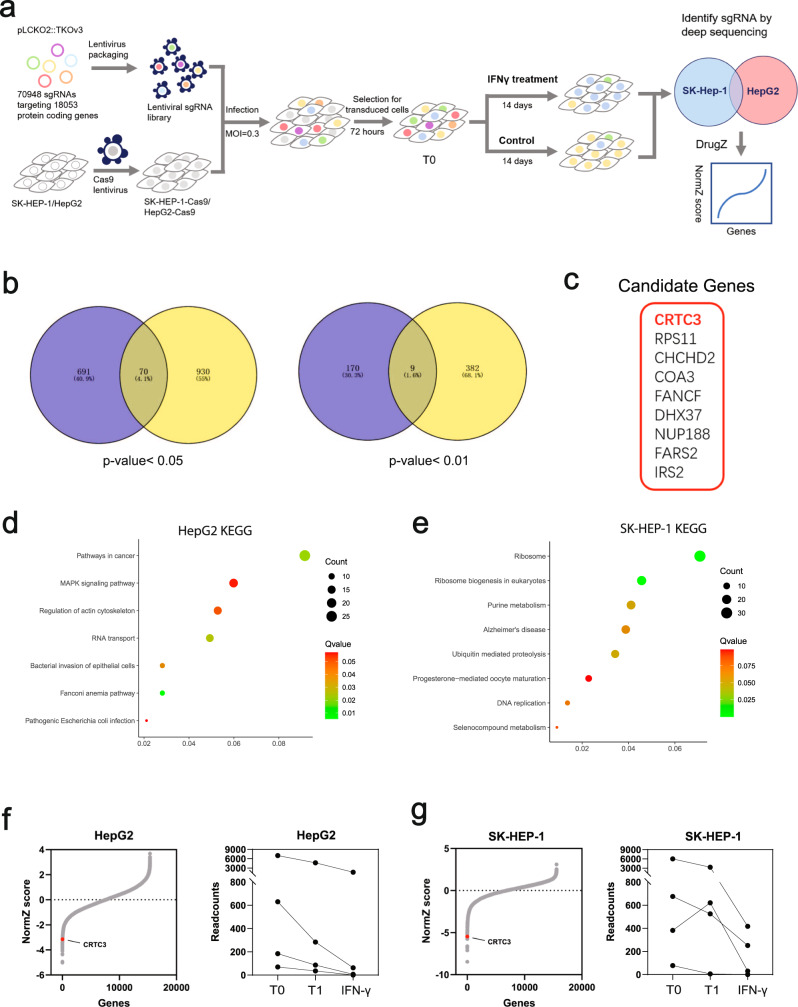
CRISPR/Cas9 screening identified driver genes that were associated with IFN-γ resistance in HCC. **a** General workflow of the CRISPR/Cas9 knockout library screening in this study. **b** Venn diagrams displaying CRISPR screening results. There were 70 significantly expressed resistance inducers, defined as *P* < 0.05 and 9 top resistance inducers, defined as *P* < 0.01 when intersecting HepG2 and SK-Hep-1 cell lines. **c** Nine top resisters identified as candidate genes of the screening. **d**, **e** KEGG enrichment analyses for significant gene hits in HepG2 cells and SK-HEP-1 cells. **f**, **g** The synergistic or suppressing effects on drug treatment were determined using the DrugZ algorithm and NormZ scores. The abundance of sgRNAs as the readout of library screening was analyzed using Bowtie2. CRTC3 was a significant depleted gene hit in both cell lines. The amount of sgRNA targeting CRTC3 was markedly decreased in CRTC3-KO HCC cells when treated with IFN-γ, compared to the control (T1).

The general workflow of CRISPR/Cas9 screening was as shown in Fig. 1a. HepG2 and SK-Hep-1 cell lines that had been subjected to IFN-γ or vehicle treatments were used for screening. Normalized Z-scores (NormZ) measured by DrugZ were used to identify genes that were associated with sensitivity or resistance to IFN-γ. Knockout of resistant genes (resisters) sensitized cells to treatment and cells carrying sgRNAs targeting resisters were less abundant in the final pool (NormZ <0). Knockout of sensitive genes (sensitizers) enhanced resistance to treatment while cells carrying sgRNAs targeting sensitizers were abundant in the final pool (NormZ >0). After removing sgRNAs of read ≤50 in startup of CRISPR screening (Day 0) and intersecting the data between the cell lines, there were 70 significant resisters defined as *P* < 0.05 and 9 top resisters defined as *P* < 0.01 (Fig. 1b, c). KEGG enrichment analysis showed that significant resisters of IFN-γ were enriched in the mitogen-activated protein kinases (MAPKs) signaling pathway and pathways in cancer in HepG2 cells, and were enriched in the ribosome, ribosome biogenesis in eukaryotes and selenocompound metabolism pathways in SK-Hep-1 cells (Fig. 1d, e). In our previous study, we performed CRISPR/Cas9-based screening of Hep3B and SNU-398 cell lines treated with sorafenib and erastin, two reported ferroptosis inducers targeting system xc-, and identified that significant resisters of both drugs were enriched in the selenocompound metabolism pathway [19]. This pathway is involved in the biosynthesis of GPx4, which is a selenium-containing enzyme and a central regulator of ferroptosis [20]. Among the 9 top resisters, CRTC3 and RPS11 were the two most significant genes. The absolute NormZ values of both genes were ranked at the front in each cell line. The TKOv3 library we used for screening designed 4 different sgRNAs targeting each gene. After removing sgRNAs of read ≤50, there were only two sgRNAs left for RPS11, while CRTC3 had all four sgRNAs, which represented a higher data quality of CRTC3 in this screening. Finally, CRTC3, a transcriptional coactivator involved in cAMP-PKA pathway and lipid metabolism [15], was identified to be the top resister in this study. The abundance of cells carrying sgRNA specific for CRTC3 was markedly decreased after IFN-γ treatment, compared to vehicle (control), suggesting a significant sensitizing role of CRTC3 knockout to IFN-γ treatment in HCC cells (Fig. 1f, g).

**Fig. 2:**
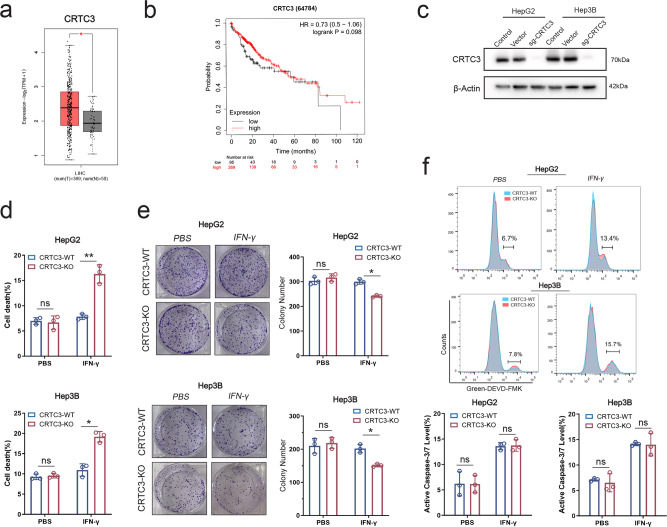
In vitro assays confirmed that CRTC3 significantly induced resistance to IFN-γ treatment in HCC. **a** Expression levels of CRTC3 mRNA in tumor and normal tissues were revealed by analysis of the TCGA-LIHC database. **b** Kaplan–Meier curves did not reveal significant correlations between CRTC3 expressions and HCC patient survival outcomes. **c** Western blotting confirmed the knockout efficacies of CRTC3 in HepG2 and Hep3B cell lines. **d** Flow cytometry analyses were performed to reveal cell death under conditions of CRTC3 knockout and IFN-γ treatment (100 ng/mL). CRTC3 knockout alone did not have significant effects on cell death. Simultaneous treatment with IFN-γ induced CRTC3-KO HepG2 and Hep3B cell deaths, compared to the control (*n* = 3). **e** Colony formation assays were used to assess long-term cell viability after treatment with IFN-γ (100 ng/mL; *n* = 3). **f** Caspase 3/7 assay demonstrated comparable caspase activities induced by IFN-γ (100 ng/mL) in CRTC3-WT and CRTC3-KO HCC cell lines (*n* = 3). However, this effect was not enhanced by CRTC3 knockout. *P < 0.05; **P < 0.01; ***P < 0.001; Student’s *t* test.

Analysis of CRTC3 mRNA levels using The Cancer Genome Atlas Liver Hepatocellular Carcinoma (TCGA‑LIHC) database revealed that HCC tissues exhibited significantly higher mRNA levels of CRTC3, compared to normal tissues (Fig. 2a). However, CRTC3 levels did not correlate with patient survival (Fig. 2b). To verify the results of our screening, we generated CRTC3-KO HCC cell lines (HepG2 and Hep3B) using the CRISPR/Cas9 knockout system and confirmed the knockout efficiency by western blotting (Fig. 2c, WB original Fig. 2c Rep1–3). In both short-term 7-AAD and long-term colony formation assays, cell survival was not significantly affected by CRTC3 knockout alone. However, IFN-γ was shown to induce significant cell death in both CRTC3-KO HCC cell lines, compared to control (Fig. 2d, e), indicating that CRTC3 induces resistance to IFN-γ treatment. Since IFN-γ was reported to induce apoptosis in tumor cells, we further performed Caspase 3/7 assay to verify whether CRTC3 knockout sensitized HCC cells to treatment by inducing apoptosis. We found that IFN-γ induced comparable mild apoptosis in CRTC3-WT and CRTC3-KO HCC cells, however, this effect was not enhanced by CRTC3 knockout (Fig. 2f).

### CRTC3-regulated ferroptosis-related gene expressions

**Fig. 3:**
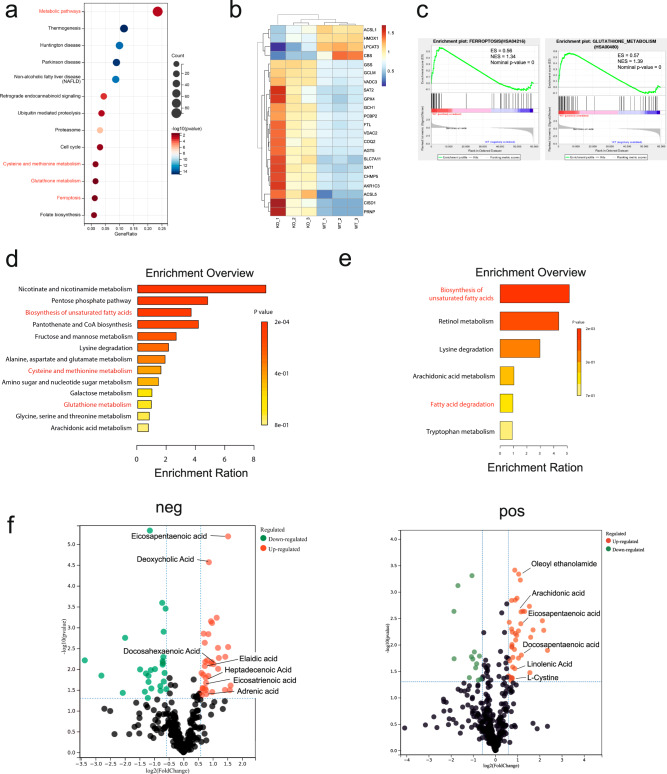
Comprehensive RNA-seq and metabolomic analyses revealed that CRTC3 regulates unsaturated fatty acid metabolism and ferroptosis. **a** KEGG enrichment analyses of differentially expressed genes following CRTC3 knockout. The metabolic pathways, ferroptosis, and its related pathways were significantly enriched. **b** Heatmaps of gene hits in the ferroptosis gene set. ACSL5, SLC7A11, and GPx4 were upregulated, and LPCAT3 was downregulated following CRTC3 knockout. **c** GSEA revealed that ferroptosis and GSH metabolism gene sets were positively correlated with CRTC3 knockout. **d** MSEA of differentially abundant metabolites revealed significant enrichments in unsaturated fatty acids biosynthesis pathways and ferroptosis-related pathways. **e** MSEA of differentially abundant lipids revealed significant enrichments in pathways of unsaturated fatty acid biosynthesis and fatty acid degradation. **f** Volcano plots for altered metabolites in negative ion modes and positive ion modes. A wide range of PUFAs were markedly increased following CRTC3 knockout.

To identify the global transcriptome affected by CRTC3 knockout, RNA sequencing (RNA-seq) analysis was performed in Hep3B-CRTC3-KO and Hep3B-vehicle cells. KEGG analysis revealed that differentially expressed genes were markedly enriched in metabolic pathways (HSA01100). In addition, ferroptosis (HSA04216) and its related pathways, including the cysteine and methionine metabolism pathway (HSA00270) and the glutathione metabolism pathway (HSA00480) were also enriched (Fig. 3a). When mapping into the GSEA database of gene hits in the ferroptosis gene set (64 genes in total), there were 18 unregulated and 4 downregulated differentially expressed genes shown in the heatmap analysis (Fig. 3b), among which, Acyl-CoA synthetase long-chain family member 5 (ACSL5) and SLC7A11 were the two most significantly upregulated genes, and recombinant lysophosphatidylcholine acyltransferase 3 (LPCAT3) was the most significantly downregulated gene according to fold changes. Besides SLC7A11, GPx4, another key regulator and protector of ferroptosis, functioning as a direct LPO scavenger [10, 11], was also upregulated with significant differences. GSEA revealed that ferroptosis and glutathione metabolism-related genes were positively correlated with CRTC3 knockout (Fig. 3c).

### CRTC3-regulated unsaturated fatty acid metabolism and ferroptosis

To investigate the biological effects of CRTC3 knockout on metabolites, a metabolomics study was conducted in Hep3B-CRTC3-KO and Hep3B-vehicle cells. The MSEA of differentially abundant metabolites revealed significant enrichments of pathways involved in unsaturated fatty acids biosynthesis following CRTC3 knockout. Ferroptosis-related pathways, including cysteine and methionine metabolism pathways as well as the glutathione metabolism pathway were also enriched (Fig. 3d), consistent with RNA sequencing analysis. The abundance of PUFAs determines cell susceptibility to ferroptosis [10, 11], therefore, we focused on lipid metabolism, especially the variations of PUFAs following CRTC3 knockout. The MSEA of differentially abundant lipids revealed significant enrichments in pathways of unsaturated fatty acid biosynthesis and fatty acid degradation (Fig. 3e). Various PUFAs, including AdA and AA were markedly elevated (Fig. 3f). Recently, Liao et al. reported that certain types of PUFAS, especially AA, can cooperate with IFN-γ to induce tumor cell ferroptosis [18], which is consistent with our findings and might be the underlying mechanisms interacting CRTC3 with ferroptosis and IFN-γ sensitization. The RNA sequencing analysis revealed the downregulation of LPCAT3, a key enzyme involved in PUFAs esterification and ferroptosis [16, 17]. Since lipid peroxidation mainly occurs on esterified PUFAs, we inferred that this might be another compensatory mechanism to protect CRTC3-KO HCC cells from ferroptosis. Consistent with elevated SLC7A11 expressions, as revealed by RNA sequencing analysis, we established that the abundance of cystine was increased in CRTC3-KO HCC cells, indicating enhanced system xc− activities.

### CRTC3 enhanced ferroptosis-related drug resistance in HCC cells

**Fig. 4:**
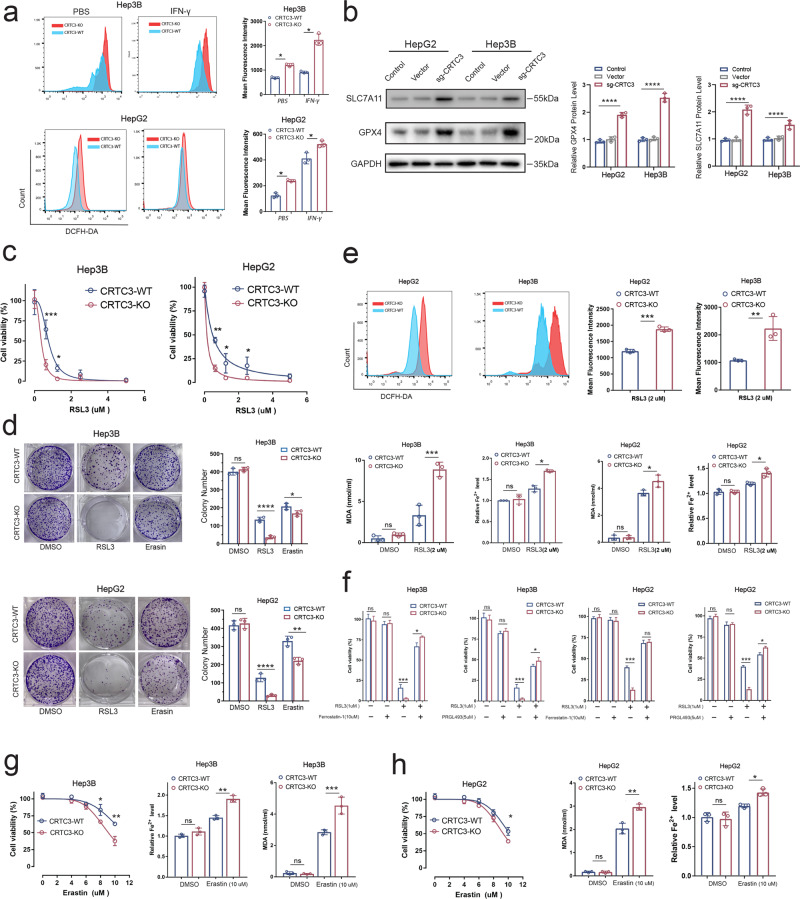
CRTC3 depletion enhanced sensitivity to treatment by inducing ferroptosis. **a** Flow cytometry analyses revealed higher baseline levels of ROS in Hep3B and HepG2 cells (*n* = 3), which were further increased by IFN-γ treatment (250 ng/mL). **b** Western blotting (replicated for three times) revealed increased expressions of SLC7A11 and GPx4 in both Hep3B and HepG2 cell lines following CRTC3 knockout. **c** CRTC3-KO and CRTC3-WT Hep3B and HepG2 cells were treated with various doses of RSL3, after which CCK-8 assays were used to assess short-term cell viability (*n* = 6). **d** Colony formation assays were used to assess long-term cell viability after treatment with RSL3 (500 nM; *n* = 3). **e** RSL3 (1 μM) treatment for 24 h markedly elevated MDA, iron and ROS levels in CRTC3-KO Hep3B and HepG2 cells, compared to control cells (*n* = 3). **f** CRTC3-KO and CRTC3-WT Hep3B and HepG2 cells were treated with RSL3 (1 μM) with or without a ferroptosis inhibitor (Ferrostatin-1 (10 μM)) and an ACSL4 inhibitor (PRGL493 (5 μM)). CCK-8 assays were used to assess the inhibition of cell proliferation (*n* = 6). **g**, **h** CRTC3 knockout sensitized HCC cells to erastin (*n* = 6), with amplified MDA and ion levels (*n* = 3). *P < 0.05; **P < 0.01; ***P < 0.001; Student’s *t* test.

Next, we determined whether CRTC3 knockout contributed to drug sensitization by inducing ferroptosis. The CRTC3-KO Hep3B and HepG2 cells exhibited higher baseline levels of reactive oxygen species (ROS), which were further elevated by IFN-γ treatment (Fig. 4a). RNA-seq analysis revealed elevated levels of ferroptosis-related gene hits. Western blotting confirmed that SLC7A11 and GPx4 were upregulated after CRTC3 knockout, which could be compensatory mechanisms for HCC cells under oxidative stress (Fig. 4b, WB original Fig. 4b Rep1-3). Further, we evaluated cell sensitivity to a typical ferroptosis inducer (RSL3), which inhibits GPx4. In both short-term and long-term proliferation assays, CRTC3 knockout significantly enhanced the suppressive activities of RSL3 in HCC cell lines (Fig. 4c, d). Then, we used the Hep3B and HepG2 cell lines and confirmed that iron, ROS and malondialdehyde (MDA) levels were significantly elevated by RSL3 treatment of CRTC3-KO HCC cells (Fig. 4e). To specify the types of cell death, we used a ferroptosis inhibitor (Ferrostatin-1) and found that CRTC3-KO and CRTC3-WT HCC cell death upon RSL3 treatment were markedly inhibited by Ferrostatin-1, and the inhibitory degree was much higher in the CRTC3-KO group (Fig. 4f). Acyl-CoA synthetase long-chain family member 4 (ACSL4), a key enzyme for PUFAs esterification, is essential for lipid peroxidation and ferroptosis [21]. To determine whether ferroptosis was directly enhanced by increased abundance of PUFAs in CRTC3-KO HCC cells, assays involving the ACSL4 inhibitor (PRGL493) showed that CRTC3-WT and CRTC3-KO HCC cell death upon RSL3 treatment could be rescued by PRGL493, and the rescue degree was more significant in CRTC3-KO HCC cells (Fig. 4f). Erastin is another typical ferroptosis inducer that targets system xc−. We confirmed that CRTC3 knockout sensitized HCC cells with amplified MDA and ion levels to erastin (Fig. 4d, g, h). These experiments confirm that CRTC3 plays a major role in regulating ferroptosis and ferroptosis-related drug resistance in HCC.

### CRTC3 depletion sensitized HCC cells to sorafenib

**Fig. 5:**
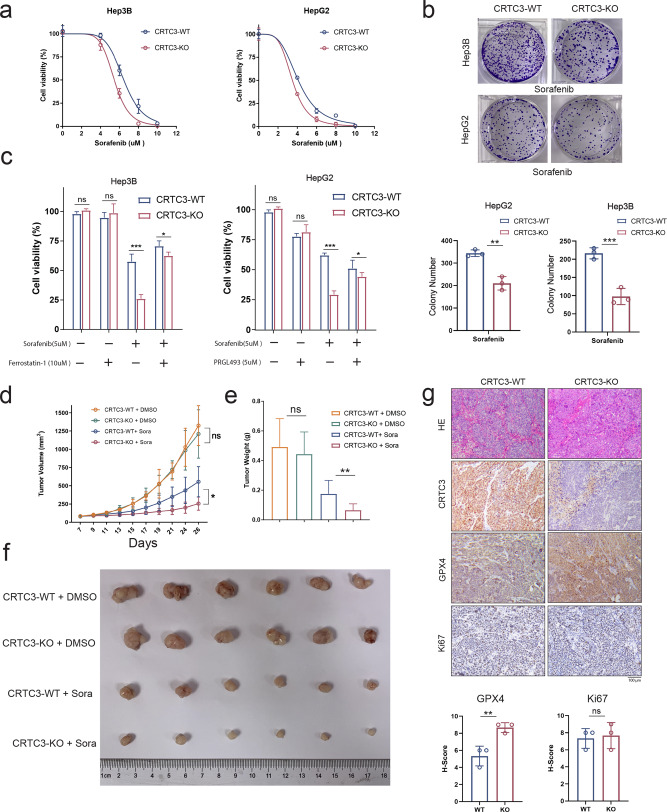
In vitro and in vivo experiments confirmed that CRTC3 knockout sensitizes HCC cells to sorafenib treatment by inducing ferroptosis. **a** CRTC3-KO, CRTC3-WT Hep3B, and HepG2 cells were treated with various doses of sorafenib, after which CCK-8 assays were used to assess short-term cell viability (*n* = 6). **b** Colony formation assays were performed to assess long-term cell viability after treatment with sorafenib (2.5 μM) (*n* = 3). **c** CCK-8 assays showed that inhibition of cell growths after sorafenib treatment (5 μM) were rescued by Ferrostatin-1 (10 μM) and PRGL493 (5 μM) treatment of CRTC3-KO Hep3B cells (*n* = 6). **d** Changes in tumor volumes over time of mice implanted with CRTC3-KO or CRTC3-WT Hep3B cells and treated with or without sorafenib (*n* = 6). **e**, **f** Measures of tumor weights and tumor volumes at the endpoint of the study (*n* = 6). **g** Representative images (six random visual fields) of HE, Ki-67, CRTC3, and GPx4 staining of tumor samples from CRTC3-KO and CRTC3-WT groups (without treatment). Immunohistochemical scores (*n* = 3) showed that GPx4 expressions were significantly elevated after CRTC3 knockout. Scale bar: 100 μm. *P < 0.05; **P < 0.01; ***P < 0.001; Student’s *t* test.

Sorafenib, a commonly used target drug for HCC treatment, was reported as a ferroptosis inducer [22]. Evaluation of sensitizing effects of CRTC3 depletion to sorafenib treatment revealed significantly enhanced anti-tumor effects in CRTC3-KO HCC cells (Fig. 5a, b), which were separately rescued by Ferrostatin-1 and PRGL493 treatments (Fig. 5c). In vivo, we subcutaneously implanted BALB/c Node mice with Hep3B-CRTC3-KO cells and Hep3B-vehicle cells, and treated them with sorafenib or vehicle when tumors were palpable. Tumor growths in CRTC3-KO cell-bearing mice were significantly inhibited by sorafenib treatment, compared to the vehicle (Fig. 5d, e). Differences in mice body weights between control and sorafenib treatment groups were not significant. Immunohistochemistry (IHC) analyses of tumor tissues confirmed that CRTC3 knockout enhanced the expressions of GPx4 in vivo (Fig. 5f). These results show the sensitizing effects of CRTC3 knockout to ferroptosis inducers, and provide a clue for overcoming drug resistance to sorafenib treatment in HCC.

## Discussion

Immunotherapy has shifted the HCC treatment paradigm. Immune checkpoint inhibitors have been recommended as preferred regimens by various guidelines. However, since only a percentage of patients are responsive, there is a need to identify the factors that affect its efficacy [4, 9]. Two separate studies reported that IFN-γ from ICIs-activated CD8 + T cells sensitized tumor cells to ferroptosis, implying that targeting ferroptotic pathways constitutes a tumor therapeutic approach when combined with ICIs [8, 18]. In this study, we established that CRTC3 induces resistance to IFN-γ treatment, while CRTC3 knockout promoted ferroptosis induction after IFN-γ treatment. Our findings elucidate a novel role of CRTC3 in regulating ferroptotic cell death, and presented a potential target of the CRTC3 pathway for cancer treatment.

CRTC3 are commonly expressed in fat [15], liver [23, 24], and bone marrow-derived immune cells [25, 26]. In basal states, CRTC3 is phosphorylated by Salt Inducible Kinases (SIKs) and other energy as well as stress-sensing AMP-activated protein kinase (AMPK) family members [15, 27]. Under hormonal and environmental stimulation, cAMP induces PKA-mediated phosphorylation and inhibition of SIKs, leading to CRTC3 dephosphorylation and activation [14, 15]. By analyzing the TCGA‑LIHC database, we found that high CRTC3 expression predicted a trend toward better survival in HCC, the reason of which is difficult to define. For the physiological role of CRTC3, previous reports mainly focused on energy balance and lipid metabolism [15]. Besides, other studies reported that CRTC3 controls the interconversion of classically activated macrophages (M1) and regulatory macrophages (M2) [26], and mediates mitochondrial biogenesis and stress response when the mitochondria are stressed in liver cells [24]. However, studies focusing on CRTC3 in cancer are limited. Only a few reports showed that CRTC3 promoted melanogenesis by regulating the expression of some key melanogenesis-related genes in melanoma [28]. In this study, we generated CRTC3-KO HCC cells and confirmed that cell survival was not significantly affected by CRTC3 knockout alone. CRTC3 had no significant impact on tumor growth in HCC cells both in vitro and in vivo. Nevertheless, the relationship between CRTC3 expression and patient survival should be confirmed using our clinical data, and this is a limitation of this study.

For the first time, we reported that CRTC3 regulates ferroptosis by altering tumor cell lipid patterns and controlling the abundance of PUFAs in HCC. Various PUFAs are prime substrates for lipid peroxidation and are involved in ferroptosis [16, 17]. Inhibition of enzymes that are essential for PUFAs biosynthesis or esterification, such as ACSL4, confers significant protective effects against ferroptosis [21]. Displacement of PUFAs by mono-unsaturated fatty acids (MUFAs) from phospholipids in the plasma membrane inhibits ferroptosis [29]. Thus, the abundance of PUFAs is closely correlated with lipid peroxidation degree and determines cell susceptibility to ferroptosis. Besides, ferroptosis occurs via impairment of LPO clearance [10, 11]. GPx4, the central regulator of ferroptosis, functions as a direct LPO scavenger [20]. GSH is a tripeptide that consists of cysteine, glutamate as well as glycine and is a co-factor that GPx4 uses to eliminate LPO from cells [20]. The biogenesis of GSH is dependent on system xc−, which imports cystine to synthesize intracellular cysteine [30, 31]. Thus, impairment of system xc- dysregulates LPO clearance, resulting in ferroptosis. Under CRTC3 knockout conditions, various PUFAs were markedly elevated in HCC cells, promoting lipid peroxidation and LPO accumulation. As a compensatory mechanism, HCC cells upregulated SLC7A11 and GPx4 expressions for LPO clearance. Since sorafenib and IFN-γ target system xc−, CRTC3 knockout facilitates sorafenib- or IFN-γ-induced ferroptosis of HCC cells. Consistent with our findings, Liao reported that AA cooperated with IFN-γ to induce ferroptosis in melanoma cell lines, and AA supplementation enhanced the anti-tumor effect of PD-L1 blockade in immunocompetent mice [18]. Targeting lipid metabolism might be a previously unappreciated approach to synergize with cancer immunotherapy.

Sorafenib is a multikinase inhibitor commonly used in the treatment of advanced HCC. It was first proposed as a ferroptosis inducer in 2013, based on the evidence that the cytotoxic effect of sorafenib on HCC cells could be significantly inhibited by iron chelators and ferrostatin-1 [22]. After that, studies repeatedly reported it as a ferroptosis inducer functioning via system xc inhibition [32, 33]. However, Zheng et al reported that sorafenib failed to induce ferroptosis in a wide range of cancer cell lines [34], indicating that the ferroptosis-inducing effect of sorafenib might be context-dependent. In this study, CRTC3 depletion definitely enhanced the cytotoxic effect of sorafenib, and the sensitizing effect could be inhibited by Ferrostatin-1 and PRGL493. We suggested that CRTC3 depletion sensitizing sorafenib treatment by induing ferroptosis. With the advent of lenvatinib and immune checkpoint inhibitors, sorafenib is less commonly used in clinics. But for target drugs monotherapy, there are hardly any drugs showing superior overall survival compared to sorafenib [4, 5]. Improving treatment efficacies of sorafenib is still of research concern. We found that CRTC3 knockout sensitized HCC cells to sorafenib by reprograming tumor cell lipid metabolism and facilitating ferroptosis, which provides a new clue for optimizing sorafenib treatment.

By analyzing the TCGA‑LIHC database, we found that tumor tissues exhibited significantly higher CRTC3 mRNA levels. However, CRTC3 protein expression was not detected in The Human Protein Atlas, as only 8 samples of HCC were examined. In the present study, we performed western blot and detected obvious CRTC3 protein expression in HepG2 and Hep3B cell lines. Nevertheless, the exact CRTC3 protein expression levels should be detected using human HCC specimens, and that’s a limitation of our study. Song reported that CRTC3 promotes obesity by attenuating sympathetic signaling and suppressing fatty acid oxidation in adipose tissues [15]. By analyzing two distinct Mexican–American cohorts, they reported that increased transcriptional activities of CRTC3 correlated with adiposity [15]. CRTC3 expression in tumor tissues might protect tumor cells from ferroptosis and result in drug resistance. Studies should investigate whether CRTC3 expressions or the degree of obesity correlates with treatment efficacies of ICIs or sorafenib in HCC.

The present study has several limitations. We did not investigate the signaling pathways responsible for the increased abundance of PUFAs following CRTC3 knockout. CRTC3 regulates lipid metabolism by communicating with catecholamine and AMPK signaling [15]. In addition, the AMPK pathway is involved in ferroptosis regulation [35, 36]. Thus, we postulated that elevations in PUFAs levels after CRTC3 knockout might be partially mediated by the AMPK pathway. In addition, CRTC3 is a coactivator of the transcription factor CREB1 [15]. The second messenger cAMP stimulates gene transcription via PKA-mediated phosphorylation of CREB1 and dephosphorylation of CRTC3. Phosphorylation of CREB1 creates a binding site for CRTC3, whose dephosphorylation and recruitment promote the efficient transcription of CREB1 target genes [15, 27]. The RNA-seq analysis revealed significantly elevated ACSL5 expression in CRTC3-KO HCC cells. We demonstrated the upregulation of ACSL5 both in mRNA (Supplementary Fig. S1a) and protein levels (Fig. 1b, WB original Supplementary Fig. S1b Rep1-3), and found a binding motif of CREB1 in the transcriptional regulatory regions of ACSL5 both by bioinformatics analysis using https://jaspar.genereg.net/ and chromatin immunoprecipitation (ChIP)-sequencing analysis using data from GSM1010808 (Supplementary Fig. S1c). ACSL5 is highly abundant in liver, and was reported to efficiently utilize multiple fatty acids to promote lipid biosynthesis and oxidation [37–39]. We inferred that ACSL5 might be a direct target of CREB1/CRTC3 and a mediator for altering lipid patterns following CRTC3 knockout in HCC cells. Further experiments are needed to make clear those underlying mechanisms. Moreover, the present study lacks in situ experiments with human specimens.

In conclusion, CRTC3 regulates PUFAs metabolism, ferroptosis and induces resistance to ferroptosis-inducing drugs. Therefore, CRTC3 is a potential therapeutic target for HCC.

## Materials and methods

### Cell lines and cell culture

Hep3B (RRID: CVCL_0326), HepG2 (RRID: CVCL_0027) and SK-Hep-1 (RRID: CVCL_0525) HCC cell lines were purchased from the American Type Culture Collection (ATCC). The cell lines were cultured in Dulbecco’s modified eagle’s medium (DMEM, Gibco) supplemented with 10% fetal bovine serum (FBS, Gibco) and 1% penicillin/streptomycin (Gibco). All experiments were performed with mycoplasma-free cells.

### Authentication of HCC cell lines

All HCC cell lines were authenticated by STR profiles. HepG2 and SK-Hep-1 cell lines were authenticated by Procell Life Science & Technology Co, Ltd (Wuhan, China). Genomic DNA was extracted from HCC cells (1 × 10^6^) using PureLink Genomic DNA Mini Kit (Life K182001, USA), which was further amplified using PowerPlex^®^18D System (Promega DC1802, USA), and sequenced by ABI3500 Genetic Analyzer (Life3500, USA). Hep3B cell lines were authenticated by BeNa Culture Collection (Beijing, China). Amplified genomic DNA was sequenced by ABI3730 XL Genetic Analyzer.

### Antibodies and reagents

Antibodies against CRTC3 (ab91654, WB, 1:1000), SLC7A11 (ab216876, WB, 1:1000), GPx4 (ab125066, WB 1:1000, IHC 1:100), and β-actin (ab8227, WB, 1:1000) were purchased from Abcam. Sorafenib (S7397), erastin (S7242), RSL3 (S8155) and Ferrostatin-1 (S7243) were purchased from Selleck Chemicals. PRGL493 (HY-139180) was purchased from MedChemExpress. IFN-γ (300-02) was purchased from PEPROTECH.

### CRISPR/Cas9 knockout library screening

The Toronto human knockout pooled library (TKOv3) comprising 70948 sgRNAs targeting 18053 protein-coding genes was a gift from Jason Moffat [40] (Addgene #125517). We amplified the library and prepared the virus in accordance with Moffat Lab protocols. Briefly, HCC cell lines were lentivirally transduced with Cas9 and mcherry coding sequences. The Cas9 stably expressing cells were confirmed by flow cytometry and western blotting. Resultant cells were transduced with a TKOv3 library at a low MOI (0.2–0.3) for 24 h and cultured in fresh DMEM containing puromycin (3 μg/mL) for 72 h. The transduced HCC cells were assigned into four groups. One group was harvested at baseline without treatment (T0), while the other groups were treated with IFN-γ (50 ng/mL) or vehicle (control) for 14 days. Finally, cells were obtained for genomic DNA extraction, which was further amplified, labeled with barcodes using NEBNext® High-Fidelity 2X PCR Master, and deep sequenced by Annoroad (Beijing, China) to assess relative enrichments of sgRNA sequences. The abundance of sgRNAs as the readout of library screening was analyzed using Bowtie2, and the synergistic or suppressing effects on drug treatment were determined using the DrugZ algorithm and NormZ scores [41].

### Establishment of CRTC3 knockout cell lines

Cas9-expressing Hep3B and HepG2 cells were transfected with lentiviral particles encoding CRTC3-sgRNA for 48 h, after which they were cultured in fresh DMEM with puromycin to obtain successfully transfected cells. Western blotting (replicated for three times) was performed to confirm the knockout efficiency. The sgRNA sequence used for CRTC3 knockout was as: CRTC3-sgRNA: 5’-TTCGGGGAACCCGCCATCAC-3’.

### Cell proliferation assays and cell death measurements by flow cytometry

For short-term cell proliferation assays (*n* = 6), we seeded 5000 HCC cells in 96-well plates and treated them with sorafenib, RSL3, erastin or vehicle for 48 h. Cell viabilities were analyzed using the CCK-8 (DOJINDO) assay, as instructed by the manufacturer. For long-term cell proliferation assays (*n* = 3), 800 cells were seeded in six-well plates and treated with IFN-γ, sorafenib, RSL3, erastin or vehicle for 10 days. Colonies were visualized by crystal violet staining, and colony numbers counted using the ImageJ software (1.48 v). For cell death analysis (*n* = 3), HCC cells were treated with IFN-γ (100 ng/ml) or vehicle (control) for 48 h, then resuspended in PBS containing 1 μg/ml 7-Aminoactinomycin D (7-AAD) for 15 min, and directly run on a flow cytometer. Apoptosis and active Caspase 3/7 levels were measured using the Active Caspase 3/7 Staining Kit (ab284532, Abcam) according to the manufacturer’s protocols (*n* = 3), and analyzed by flow cytometry.

### Western blotting assay

Total proteins in the cell lysate were extracted using the RIPA lysis buffer (KeyGEN Biotech), and further separated by SDS-PAGE. Then, samples were transferred onto PVDF membranes and incubated with indicated antibodies. Signals were determined using the Omni-ECL™Femto Light Chemiluminescence Kit (SQ201, EpiZyme), according to the manufacturer’s instructions. All western blotting assays were replicated for three times.

### RNA sequencing and untargeted metabolomics

We performed RNA sequencing analysis to assess the global transcriptome that had been affected by CRTC3 knockout using CRTC3-KO and CRTC3-WT Hep3B cells (three biological replicates). Genes with adjusted p value (padj) <0.05 and fold change ≥1.5 were defined as differentially expressed, and their enrichments in Kyoto Encyclopedia of Genes and Genomes (KEGG) pathways were determined using the clusterProfiler R package and by Gene set enrichment analysis (GSEA) using GSEA software (4.1.0). Untargeted metabolomics analyses were performed to determine the biological impacts of CRTC3 knockout on metabolites using CRTC3-KO and CRTC3-WT Hep3B cells (five biological replicates). Differentially expressed metabolites were determined using the thresholds of <0.05 and fold change ≥1.5. The metabolite set enrichment analysis (MSEA) was performed using MetaboAnalyst 5.0 to determine the enrichments of differentially expressed metabolites. Volcano plots were used to filter metabolites of interest based on log2 (FoldChange) and −log10 (*P* value). RNA sequencing and Untargeted metabolomics analyses were performed as previously reported [19].

### Measurement of ferroptosis

The MDA concentrations were analyzed using a Lipid Peroxidation (MDA) Assay Kit (*n* = 3)(#BC0025, Solarbio), while iron concentrations were analyzed using an Iron Assay Kit (*n* = 3) (#BC4355, Solarbio), according to manufacturer’s instructions. To assess ROS levels (*n* = 3), HCC cells were cultured with ferroptosis inducers (sorafenib, RSL3, and IFN-γ) or vehicle for 24 h, after which the cells were stained using 2’,7’-dichlorodihydrofluorescein diacetate (DCFH-DA) (KeyGEN BioTECH). ROS levels were assayed and analyzed by flow cytometry and the FlowJo software (V10).

### Xenograft

Animal assays were performed in accordance with the Institute of Biophysics, Chinese Academy of Science’s Policy on Care and Use of Laboratory Animals. We purchased 6–8 week-old female BALB/c Nude mice from HFK BIOSCIENCE (Beijing). Hep3B-vehicle/Hep3B-CRTC3-KO cells were subcutaneously implanted (1 × 10^7^ cells in 200 μL PBS) into mice (upper flank). Tumor-bearing mice were randomized into two groups (*n* = 6 per group) and orally treated with sorafenib (50 mg/kg, every other day) for 2–3 weeks. Tumor volumes and mice weight were monitored. Tumor volumes were quantified using the modified ellipsoidal formula, tumor volume = (length × width^2^)/2.

### Immunohistochemical staining

Tumor tissues were obtained from CRTC3-KO or CRTC3-WT mice groups without treatment (*n* = 3). Immunohistochemical staining of CRTC3 (1:100) and GPx4 (1:100) was performed on HCC samples using Immuno-Histo Stainer. The immunostaining score was determined as: percentage score × intensity score. Comparisons of GPx4 expressions between the groups were analyzed by nonpaired Student’s *t* test.

### Statistical analysis

Data are shown as mean ± standard deviation (SD). Comparisons between groups were performed by nonpaired Student’s *t* test (GraphPad Prism 8). The Cancer Genome Atlas Liver Hepatocellular Carcinoma (TCGA‑LIHC) database was used to analyze gene expression and patients' survival. CRTC3 expression analysis was performed using GEPIA2 (http://gepia2.cancer-pku.cn/). Kaplan–Meier curves were plotted to assess survival outcomes using R 3. 6. 1 package, splitting patients by lower quartile of CRTC3 expression. *P* ≤ 0.05 was the threshold for statistical significance.

### Supplementary information

Supplementary material

WB original

## Data Availability

Data from sgRNA sequencing, RNA-seq, and metabolomics were deposited in the supplementary file of the paper (GSE206905, GSE206906, and GSE206907).
