# Correction: In vitro CRISPR screening uncovers CRTC3 as a regulator of IFN-γ-induced ferroptosis of hepatocellular carcinoma

**DOI:** 10.1038/s41420-023-01745-y

**Published:** 2023-12-18

**Authors:** Li Li, Tao Xing, Yiran Chen, Weiran Xu, Bo Fan, Gaoda Ju, Jing Zhao, Li Lin, Cihui Yan, Jun Liang, Xiubao Ren

**Affiliations:** 1 Department of Immunology, Tianjin Medical University Cancer Institute and Hospital, National Clinical Research Center for Cancer, Tianjin, China; 2 Tianjin’s Clinical Research Center for Cancer, Tianjin, China; 3 Key Laboratory of Cancer Immunology and Biotherapy, Tianjin, China; 4 Department of Oncology, Peking University International Hospital, Beijing, China; 5 Key Laboratory of Carcinogenesis and Translational Research (Ministry of Education), Department of Oncology, Peking University Cancer Hospital and Institute, Beijing, China; 6 Department of Radiation Oncology, Fujian Medical University Cancer Hospital, Fujian Cancer Hospital, Fuzhou, China; 7 Department of Oncology, Beijing Tiantan Hospital, Capital Medical University, Beijing, China; 8 Beijing Chao-Yang Hospital, Beijing, China; 9 Department of Pathology and Neuropathology, University Hospital Tübingen, Tübingen, Germany

**Keywords:** Cancer therapeutic resistance, Cancer therapeutic resistance

Correction to: *Cell Death Discovery* 10.1038/s41420-023-01630-8, published online 4 September 2023

In this article a grant number has been added:

This study was supported by grants from the Natural Science Foundation of China (grant No. 82202882, 81872166, U20A20375), Beijing Natural Science Foundation (grant No. 7232227), Foundation of Peking University International Hospital (grant no.YN2022ZD01, YN2021ZD03), and Beijing Xisike Clinical Oncology Research Foundation (grant no. Y-BMS2019-015).

The original article has been corrected.

