# Supplementary material for: In vitro CRISPR screening uncovers CRTC3 as a regulator of IFN-γ-induced ferroptosis of hepatocellular carcinoma
Source: Cell Death Discov. 2023 Sep 4;9:331. doi: 10.1038/s41420-023-01630-8 (PMC10477178; doi:10.1038/s41420-023-01630-8)
Supplement: Supplementary file 1 — Supplementary material [file 41420_2023_1630_MOESM1_ESM.pdf]

***In vitro* CRISPR screening uncovers CRTC3 as a regulator of IFN-γ-induced ferroptosis of hepatocellular carcinoma**

Li Li[1,2,3,4,*], Tao Xing[5,*], Yiran Chen[6,*], Weiran Xu[7,*], Bo Fan[8], Gaoda Ju[5], Jing Zhao[9], Li Lin[4], Cihui Yan[1,2,3,#], Jun Liang[4,#], Xiubao Ren[1,2,3,#]

Supplementary material and methods

Quantitative PCR

Total RNA was isolated with Trizol reagent (Invitrogen, Thermo Fisher Scientific). cDNA synthesis was performed with 1μg of total RNA using the manufacturer's protocol. Quantitative PCR (qPCR) was performed on cDNA containing TB green reagent (RR820A; TaKaRa, Tokyo, JAPAN) and specific primers on a Real-time PCR machine (qTOWER3G, Analytik Jena, Jena, Germany) (n=3). All results were normalized to β-actin mRNA levels. Gene expression levels were measured using the ΔΔCt method. Sequences of primers are as follows:

ACSL5 forward: TCCTGACGTTTGGAACGGC

ACSL5 reverse: CTCCCTCAATCCCCACAGAC

β-actin forward: GAGAAAATCTGGCACCACACC

β-actin reverse: GGATAGCACAGCCTGGATAGCAA

Supplementary figures

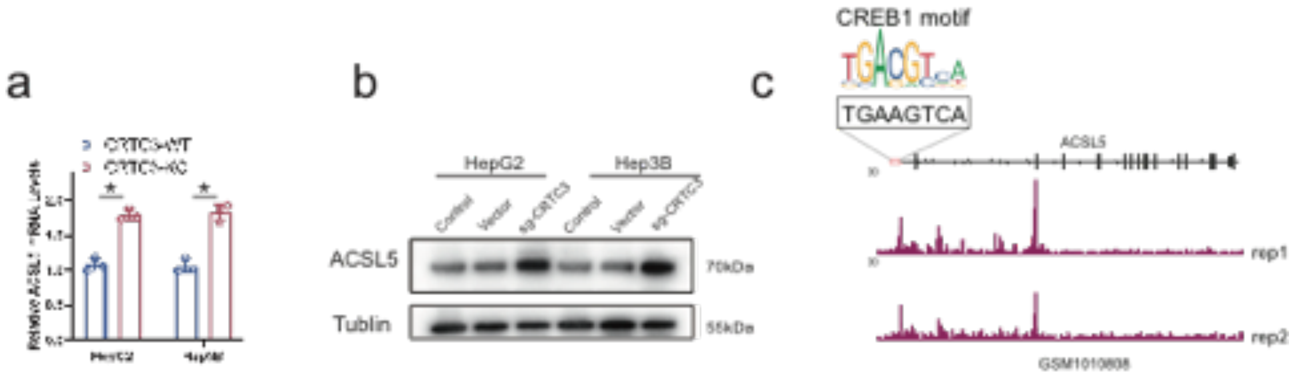

Fig. S1: Bioinformatics and ChIP-sequencing analysis revealed ACSL5 as a direct target of CREB1.

a. qPCR analysis showed enhanced ACSL5 expression in mRNA levels following CRTC3 knockout in both Hep3B and HepG2 cell lines (n=3). **b.** Western blotting revealed enhanced ACSL5 expression in protein levels (replicated for 3 times). **c.** Bioinformatics analysis using https://jaspar.genereg.net/ and ChIP-sequencing analysis using data from GSM1010808 found a binding motif of CREB1 in the transcriptional regulatory regions of ACSL5.
