# Supplementary material for: In vitro CRISPR screening uncovers CRTC3 as a regulator of IFN-γ-induced ferroptosis of hepatocellular carcinoma
Source: Cell Death Discov. 2023 Sep 4;9:331. doi: 10.1038/s41420-023-01630-8 (PMC10477178; doi:10.1038/s41420-023-01630-8)
Supplement: Supplementary file 2 — WB original [file 41420_2023_1630_MOESM2_ESM.pdf]

Unedited blot and gel images

Figure 2c Rep1

Band Merge

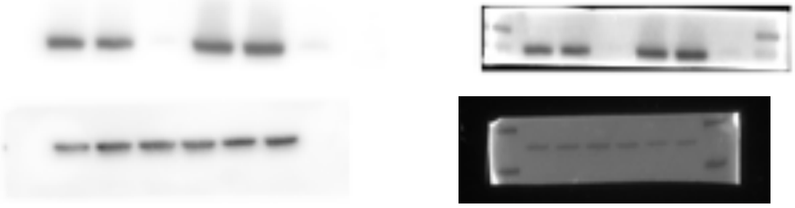

CRTC3

Actin

Figure 2c Rep2

Band Merge

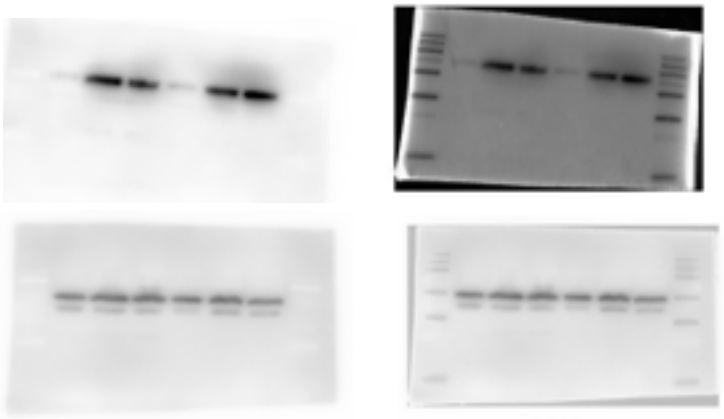

CRTC3

Tublin

Figure 2c Rep3

Band Merge

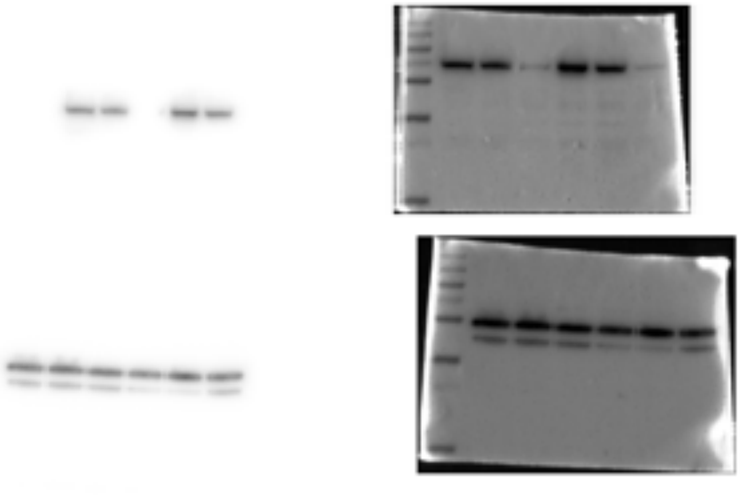

CRTC3

Tublin

Figure 4b Rep1

Band Merge

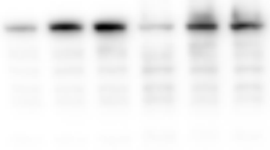

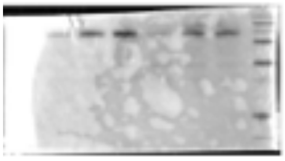

CRTC3

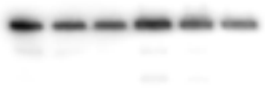

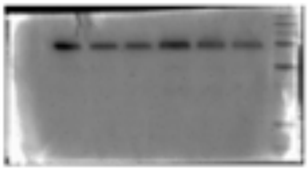

SLC7A11

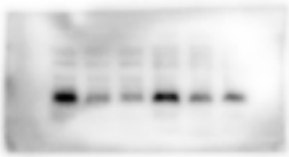

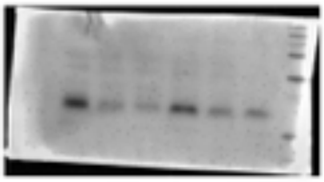

GPX4

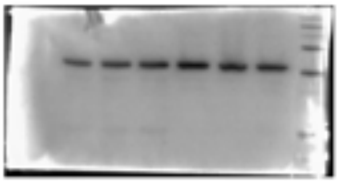

GAPDH

Figure 4b Rep2

Band Merge

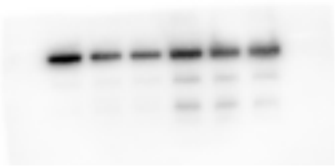

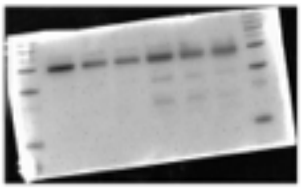

SLC7A11

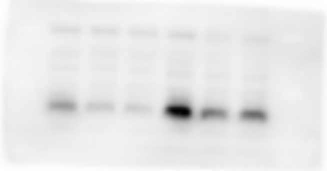

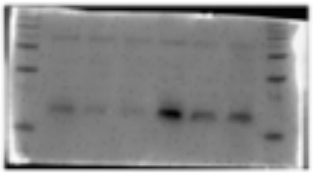

GPX4

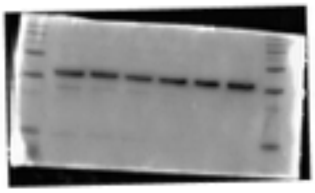

GAPDH

Figure 4b Rep3

Band

Merge

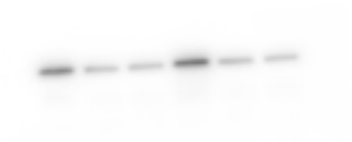

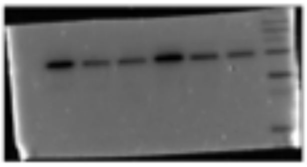

SLC7A11

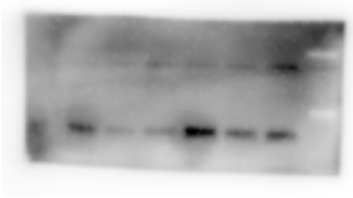

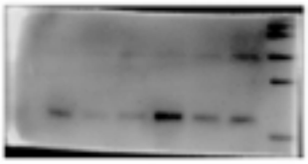

GPX4

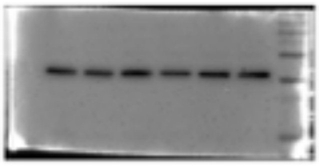

GAPDH

Figure S1b Rep1

Band Merge

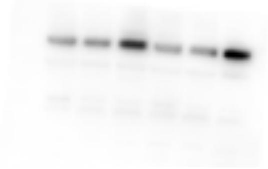

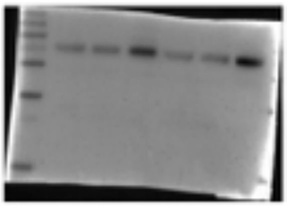

ACSL5

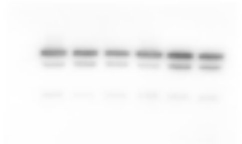

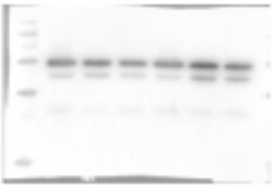

Tublin

Figure S1b Rep2

Band Merge

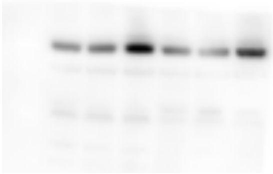

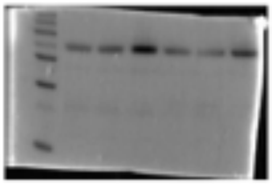

ACSL5

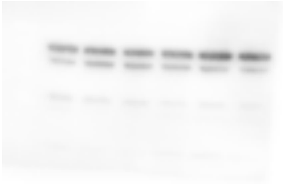

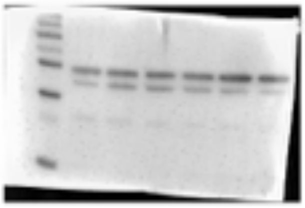

Tublin

Figure S1b Rep3

Band Merge

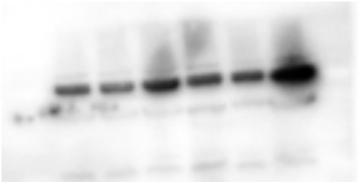

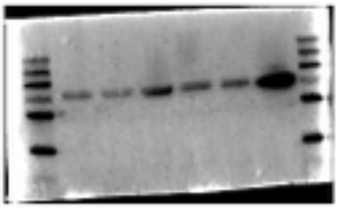

ACSL5

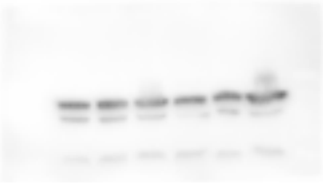

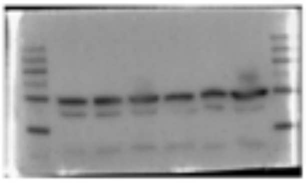

Tublin
